# Sustainable chemistry with plasmonic photocatalysts

**DOI:** 10.1515/nanoph-2023-0149

**Published:** 2023-05-30

**Authors:** Lin Yuan, Briley B. Bourgeois, Claire C. Carlin, Felipe H. da Jornada, Jennifer A. Dionne

**Affiliations:** Department of Materials Science and Engineering, Stanford University School of Engineering, Stanford, CA, 94305, USA; Department of Applied Physics, Stanford University School of Humanities and Sciences, Stanford, CA, 94305, USA; Stanford PULSE Institute, SLAC National Accelerator Laboratory, Menlo Park, CA, 95024, USA; Department of Radiology, Stanford University School of Medicine, Stanford, CA, 94305, USA

**Keywords:** hot carrier, photocatalysis, plasmonic, sustainable chemistry

## Abstract

There is a pressing global need to increase the use of renewable energy sources and limit greenhouse gas emissions. Towards this goal, highly efficient and molecularly selective chemical processes that operate under mild conditions are critical. Plasmonic photocatalysis uses optically-resonant metallic nanoparticles and their resulting plasmonic, electronic, and phononic light-matter interactions to drive chemical reactions. The promise of simultaneous high-efficiency and product-selective reactions with plasmon photocatalysis provides a compelling opportunity to rethink how chemistry is achieved. Plasmonic nanoparticles serve as nanoscale ‘antennas’ that enable strong light–matter interactions, surpassing the light-harvesting capabilities one would expect purely from their size. Complex composite structures, combining engineered light harvesters with more chemically active components, are a focal point of current research endeavors. In this review, we provide an overview of recent advances in plasmonic catalysis. We start with a discussion of the relevant mechanisms in photochemical transformations and explain hot-carrier generation and distributions from several ubiquitous plasmonic antennae. Then we highlight three important types of catalytic processes for sustainable chemistry: ammonia synthesis, hydrogen production and CO_2_ reduction. To help elucidate the reaction mechanism, both state-of-art electromagnetic calculations and quantum mechanistic calculations are discussed. This review provides insights to better understand the mechanism of plasmonic photocatalysis with a variety of metallic and composite nanostructures toward designing and controlling improved platforms for green chemistry in the future.

## 1 Introduction

What common thread unites industries spanning plastics, pharmaceuticals, cosmetics, agricultural, and fertilizers? The answer is heterogeneous catalysis. Approximately 40 % of the world’s gross domestic product relies on heterogeneous catalysts to facilitate the synthesis of valuable chemical products on a massive scale [1]. Traditional thermal-driven catalysis utilizes active transition metals, such as platinum group metals, supported on metal oxides (e.g. Al_2_O_3_, MgO, CeO_2_, etc.). Reactions typically operate under high temperatures and pressures to satisfy the thermodynamic and kinetic constraints necessary for molecular conversion. The extreme conditions required for many reactions come with significant energy consumption, greenhouse gas emission, and additional requirements for separating reaction byproducts and recycling and regenerating the catalysts. Achieving precision chemistry, where reactions are simultaneously high-yield, product selective, and free from greenhouse-gas emissions, will be critical for a sustainable future.

Light-driven reactions, powered directly by sunlight or solar-driven LEDs, hold promise for such precision chemistry. Decades of progress in the field of optics have enabled the manipulation of light at the nanoscale, resulting in efficient light-harvesting materials which have recently been shown to enable new chemical pathways. The collective oscillation of free electrons at resonant conditions with nanoscale metal antennas, known as localized surface plasmon resonances (LSPR), offers the potential to reinvent photochemical transformations by accessing potential energy surfaces through non-equilibrium excited states [2]. Using these excited-state reaction pathways which are inaccessible in thermally-driven catalysis, it is possible to achieve higher reaction rates as well as enhanced chemical selectivity, as demonstrated in propylene epoxidation [3], selective acetylene hydrogenation [4], carbon dioxide reduction [5–9] and methanol steam reforming [10, 11]. Further, such chemical transformations can be triggered at far milder temperatures and pressures than traditional thermal-driven processes.

In recent years, there has been significant growth in the field of plasmonic chemistry, with numerous excellent review articles exploring the topic [12–16]. These articles cover various aspects of plasmonic chemistry, including computational catalyst design, novel materials and hybrid composites, and applications of potential industrially relevant reactions. In contrast to these contributions, we review recent developments in sustainable chemistry applications of some of the most greenhouse-gas emitting chemical industries (ammonia synthesis, hydrogen production) and efforts towards CO_2_ utilization. The chemical industry contributes more than 3.6 × 10^13^ kg of annual global greenhouse gas emissions, corresponding to 10 % of global emissions; we therefore believe the three reactions we cover are the most suitable to tackle a transition to a sustainable, circular economy [17, 18]. We systematically summarize the efficiency and mechanism of each catalyst, while providing a fundamental understanding from the angle of light–matter interaction and surface chemistry. Our review therefore bridges the gap between fundamental plasmonic chemistry and its next-step applications in sustainable heterogeneous catalysis.

In this review, we will describe recent progress in sustainable plasmonic chemistry, focusing on three key reactions – ammonia synthesis, hydrogen production and CO_2_ reduction – and how they can address issues such as energy storage, greenhouse gas emissions alleviation, and the development of a hydrogen economy. We start by briefly introducing the decay pathways and associated lifetimes of LSPRs to understand how they convert light energy into chemical energy. We describe the design of nanostructures for both strong light–matter interactions and energy transfer near the catalyst active centers. We briefly highlight the many excellent demonstrations of plasmon catalysis utilizing metals such as Pt, Ir, Pd, and then describe recent progress in utilizing sustainable materials to replace noble metals and rare materials. We also discuss recent progress in electromagnetic simulations and quantum mechanistic calculations. Finally, we will provide a brief outlook on the field and the questions which, from our perspective, must be addressed in the future.

## 2 General mechanism for photochemical transformation

**Figure 1:**
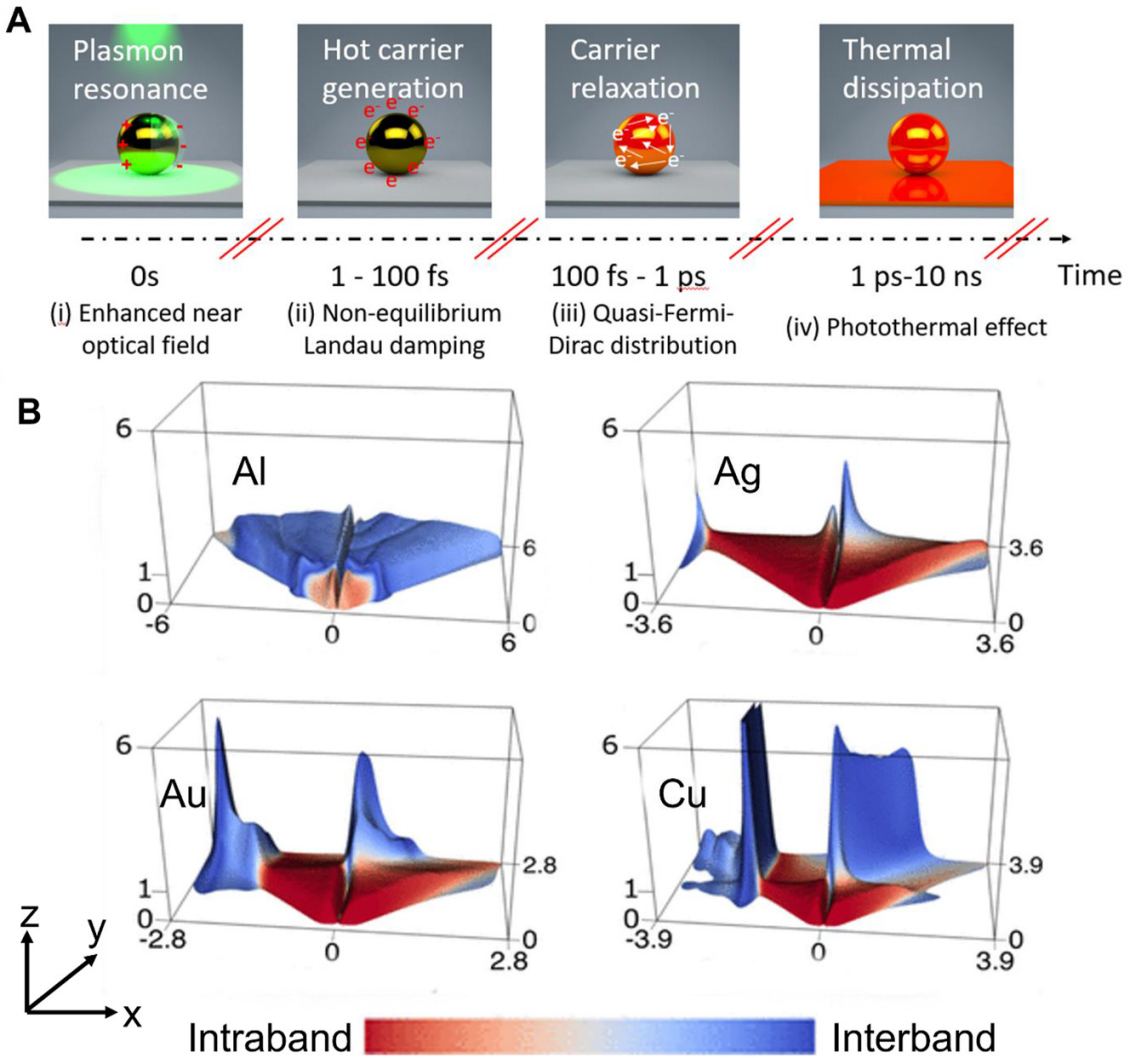
Lifetime of plasmon due to electron-hole dephasing and associated hot carrier distributions. (A) The general process of plasmon decay: (i) excitation of the localized surface plasmon resonance (LSPR) results in an enhanced interaction cross-section with the incident light from the enhanced optical near-field. (ii) Non-radiative decay through Landau damping and pumping of non-equilibrium hot carriers. (iii) Hot-carrier thermalization through electron-electron scattering. The numbers of energetic carriers (also referred to as thermalized carriers) increases. (iv) Photothermal heating from electron-phonon interaction. (B) The energy distribution of the hot carriers (*z* axis) as the function of plasmon frequency (*y* axis) and hot carrier energy (*x* axis) in Al, Ag, Au, and Cu. The red part represents the hot carrier generation through phonon-assisted Landau damping (or intraband transition), and the blue part represents the direct photoexcitation of interband transition. Reproduced from ref. [22]. Copyright 2016 American Chemical Society.

Recent focus in plasmonic photocatalysis has been dedicated to unraveling the mechanisms driving chemical reactions in the presence of photoexcited plasmonic nanoparticles [2, 19]. The current understanding in the field is that resonant light–matter interactions, based on nanoparticle geometry and material, lead to the generation of excited charge carriers [20, 21]. These carriers possess a high level of chemical reactivity. Resonant excitation of the LSPR in a plasmonic nanoparticle causes near-field electromagnetic enhancements of several orders of magnitude, essentially ‘focusing’ light to sub-wavelength spaces (Figure 1A, i). Plasmons can subsequently dephase non-radiatively through surface-assisted Landau damping, an intrinsically quantum mechanical phenomenon which generates hot electron-hole pairs within 100 fs (Figure 1A, ii). Hot carriers can undergo further relaxation through electron-electron interactions, producing a quasi-Fermi-Dirac distribution within 1 ps (Figure 1A, iii). Photochemical transformation can be facilitated by hot carriers generated from the plasmon dephasing as well as those generated by direct interband transitions, and the dynamics and time scales of these processes are distinct. If the hot carriers are not extracted to surface adsorbates (or in the form of photocurrents for other applications), the kinetic energy of the hot carriers will be dissipated as heat to the surrounding medium (Figure 1A, iv).

For photochemical transformations, hot-carrier mediated processes are considered the most efficient pathway among all energy-transfer mechanisms [23, 24]. They additionally provide higher reactivity and chemical selectivity under mild conditions than traditional thermal-driven process [3, 25]. The most well-studied plasmonic metals include Au, Ag, Al, and Cu [13, 26–28]. Energy distributions of hot carriers calculated by first-principles calculations as a function of carrier energy and plasmon frequency are outlined in Figure 1B [22]. The indirect (red) and direct (blue) components represent phonon-assisted intraband transitions and interband transitions, respectively. Hot-electron-induced reactions are well studied using small Au and Ag nanoparticles (generally <20 nm in diameter) through their hot electrons generated after their dephasing due to Landau damping [7, 29], [30], [31], [32]. The relevant energy distribution can also be achieved through the interband transition of Al [33, 34]. Recent studies of hot holes in Au and Cu show that they can be extracted to drive oxidation reactions [35–39]. Other types of plasmonic materials can be used, such as doped semiconductors, perovskites, 2D materials, conductive polymers, etc. [40] Their properties and the design of nanostructures using these materials differ in some ways from metallic nanoparticles, and we refer the interested readers to these references for more information [41–44].

A plethora of opportunities exist in tuning plasmonic structures to optimize both light harvesting properties and chemical reactivity. The electromagnetic properties of the metallic nanoparticles can be modified by changing the size, shape, composition, and dielectric medium [29]. These changes to the electromagnetic properties impact the resonance frequencies of the systems and therefore the hot carrier generation. In addition to utilizing the plasmonic metals alone as both the light-harvester and catalytic center, combining them with more traditional catalytic materials, such as transition metals or metal oxides, through nanostructured designs like antenna-reactor, core–shell, and homogeneous alloying provides additional parameter space to explore in pursuit of effective materials. In the case of alloying, a large focus has been placed on combating the inherent trade-off between plasmonic and chemically active materials. Based on ground-state electronic structure, it is often the case that noble metals (Au, Ag), which are high-performing light absorbers, have lower intrinsic reactivity than many transition group precious metals (Pd, Pt, Ir, etc.). Combining these materials can synergistically provide advantages from both groups. Meanwhile, recent work seeks to find alloys that can utilize more sustainable materials instead, such as Al, Fe, and Cu. In addition to the design of sustainable materials systems, the bulk of research in the field seeks to address chemical reactions that align with sustainability concerns, particularly in the areas of fertilizer production, greenhouse gas remediation, and high-volume chemical feedstocks.

## 3 Applications for sustainable chemistry

### 3.1 Ammonia synthesis (N_2_ reduction)

The Haber-Bosch Process is the traditional method of producing ammonia for fertilizer and emblematic of the challenge researchers must address to move the chemical industry into a sustainable future. Global population growth has been enabled specifically because of the invention of the ammonia synthesis process, contributing to ∼50 % of world population. Historically, around 78 % of the ammonia produced by the Haber-Bosch process is used in agriculture, as fertilizer to feed the world [45, 46]. Yet, despite its societal impact, the Haber-Bosch process is also responsible for a considerable carbon footprint. Traditionally, Fe-based catalysts supported on oxide are used under high temperature (300–500 °C) and pressure (100 atm) to convert N_2_ and H_2_ into NH_3_ [47, 48]. The process accounts for around 2 % of total energy from the chemical industry, and consumes 50 % of hydrogen production within the sector, leading to 3 % of annual global greenhouse gas emissions [49, 50]. There are few chemical reactions as important to the world which so critically necessitate innovation.

The fixation of dinitrogen species onto the surface of active centers is a crucial step in the reaction. The electronic structure of transition metals such as Os, Ru, and Fe makes them promising candidates to provide the active surface for this step. For instance, the first and second generations of the Haber-Bosch catalysts, which are based on Fe and Ru, respectively, rely on the Fe C7 and Ru B5 active sites for the activation of dinitrogen species [14, 51]. Plasmonic antennas offer a viable approach for improving the activation and fixation of dinitrogen species through the strong optical near-field enhancement, facilitating electronic transitions of the adsorbates by hot carriers, and traditional equilibrium and thermodynamic effects from photothermal conversion of light energy.

**Figure 2:**
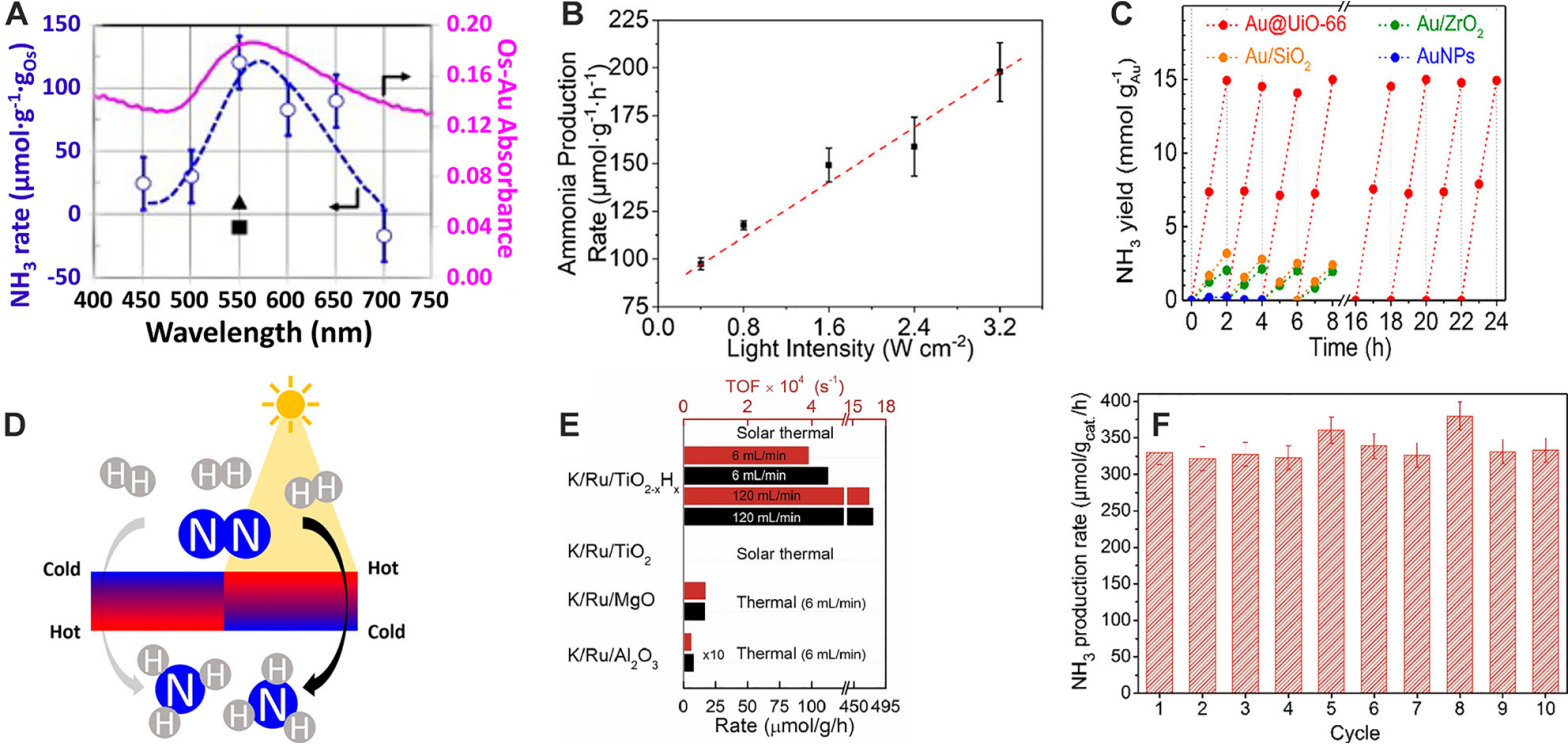
Plasmonic nitrogen fixation. (A) The wavelength-dependent reactivity (blue open dots and dashed curve) is well-correlated to the extinction spectrum (pink solid line) on a Au-Os nanoparticles for gas-phase ammonia synthesis (N_2_ + 3H_2_ → 2NH_3_). Adapted from ref. [52] with permission. Copyright 2014 Elsevier B.V. (B) Ammonia synthesis in presence of water (N_2_ + 6H^+^ + 6e^−^ → 2NH_3_) on a AuRu core-antenna nanostructure under full-spectrum irradiation at 2 atm N_2_. Adapted from ref. [53] with permission. Copyright 2019 American Chemical Society. (C) Stability test of ammonia synthesis on Au in presence of water on UiO-66 MOF. Adapted from ref. [63] with permission. Copyright 2019 American Chemical Society. (D) The plasmonic gas-phase ammonia synthesis with a commercial Ru-Cs/MgO catalyst driven by the thermal gradient. Adapted from ref. [59]. Copyright 2019 American Chemical Society. (E) Plasmonic photothermal gas-phase ammonia synthesis with a K/Ru/TiO_2−*x*_H_*x*_ catalyst. Adapted from ref. [60] with permission. Copyright 2017 Elsevier B.V. (F) Stability of a porous plasmonic CuFe for nitrogen fixation with 1 atm N_2_ in water. Adapted from ref. [62] with permission. Copyright 2020 American Chemical Society.

Early photocatalytic studies of ammonia synthesis show that light irradiation can activate N_2_ at milder conditions than the industrial Haber-Bosch process. Zeng, H., et al. demonstrated a proof of concept for photochemical ammonia synthesis by using Au-Os nanoparticles created via sputtering on a glass substrate. Gas-phase ammonia synthesis (N_2_ + 3H_2_ → 2NH_3_) is achieved at room temperature and pressure using 200 mW/cm^2^ irradiation from a solar simulator with a 450 nm cutoff filter. The study proposes that plasmons are influential to the process, since the extinction spectra of the sample matches with wavelength-dependent reactivity measurements (Figure 2A). This study represents one of the initial demonstrations of using a thin layer of Os nanoparticles as the active catalyst to drive the reaction photochemically. It is noteworthy that Os was the first transition metal historically used for traditional ammonia synthesis [52]. In an aqueous phase (N_2_ + 6H^+^ + 6e^−^ → 2NH_3_), Hu, C., et al. showed that Au-Ru nanoparticles with multiple bimetallic ratios under Xenon lamp illumination at 400 mW/cm^2^, 2 bar pressure, and room temperature can facilitate the associative alternating hydrogenation of dinitrogen species when activating the surface plasmon resonance. The research team demonstrated the plasmon-driven reactivity of the system through the linear relationship between the reactivity and the input power (Figure 2B). Moreover, their first-principle model provided mechanistic insights into the necessity of a strong electric near-field for the activation of dinitrogen species onto the Ru center [53]. Au nanoparticles on various semiconductors, such as TiO_2_, CeO_2_, and g-C_3_N_4_, achieve enhanced visible light absorption as well as charge separation at the metal-semiconductor interface for nitrogen fixation. Semiconductor materials with appropriate band structure and suitable active surfaces are inherently effective catalysts for nitrogen fixation under UV illumination. Incorporating plasmon-mediated visible light absorption with active oxygen or nitrogen defects can augment ammonia fixation, providing an additional avenue for promoting dinitrogen fixation that is distinct from traditional transition metal-based catalysts [54–57]. Supporting Au nanoparticles in the metal-organic framework (MOF) UiO-66 increases permeability owing to the large surface area of the matrix, better enabling the Au nanoparticles to activate N_2_ on their active sites. The MOF support provided excellent stability for over 24 h, a significant improvement compared to only 8 h when the same Au was supported on other metal oxides (Figure 2C). Traditionally, the Au surface of the MOF is a poor active center for the activation of dinitrogen species. However, the unique Au-MOF interface, which exhibits an enhanced surface area, displayed extraordinary reactivity and a decrease in light-induced activation [58]. There are also several studies utilizing Ru catalysts which are similar to industrial catalysts in components and structure, but now the plasmonic photothermal effect is synergistically employed to drive gas-phase ammonia synthesis. Li, X., et al. reported the LED illumination of a conventional Ru-Cs/MgO catalyst and utilized the temperature gradient caused by the plasmonic photothermal effect (Figure 2D) to achieve a reaction rate of 4464 μmol g^−1^ h^−1^ at 4.7 W/cm^2^, detecting the gas-phase conversion of N_2_ to NH_3_ in a mass spectrometer [59]. Mao, C., et al. reported a K/Ru/TiO_2−*x*_H_*x*_ catalyst at 360 °C under 300 W Xe lamp irradiation and reaching 112.6 μmol g^−1^ h^−1^(Figure 2E) [60]. The same research team also investigated a Fe-based nano-necklace/TiO_2−*x*_H_*x*_ and achieved ammonia concentration of 1939 ppm at 1 atm and 19,620 ppm at 10 atm [61]. Hou, T., et al. developed a porous Cu_96_Fe_4_ nanostructure and achieved 342 μmol g^−1^ h^−1^ at 250 mW/cm^2^ with the full spectrum of their Xenon lamp, and 242 μmol g^−1^ h^−1^ with a >400 nm filter at 200 mW/cm^2^. The catalyst also displays good stability during 10 cycles (Figure 2F) [62]. Table 1 summarizes the recent progress on N_2_ reduction. Taken together, these results show that plasmons can drive ammonia synthesis at milder temperatures and pressures than the Haber-Bosch process. Yet, further work is needed to regulate the dinitrogen activation towards the dissociative pathways and therefore boost its efficiency towards industrial scales. To identify avenues for controlling the associative hydrogenation of dinitrogen to distal and dissociation pathways, it is necessary to perform *in-situ* measurements of intermediate steps. Furthermore, high-resolution, and even atomic-resolution electron microscopy is essential to determine the active centers and their interaction evolution during the reaction. Theoretical studies are also crucial for gaining insights into the design of nanostructures, Particularly for understanding how excited-states can potentially lower the activation barrier of dinitrogen species.

**Table 1:** Summary of ammonia synthesis literature.

Nanostructure	Light	Power	Ammonia	Quantum	Mechanism	Reference
	source	density	rate	efficiency		number
Gas phase reaction: N_2_ + 3H_2_ → 2NH_3_						
Au-Os/substrate	Solar simulated (>450 nm)	200 mW/cm^2^	265 μmol g_Os_^−1^ h^−1^		Au plasmon + Os active center	[52]
Ru-Cs/MgO	White and blue LED	0–4.7 W/cm^2^	4500 μmol g^−1^ h^−1^		Light-induced thermal gradients in ruthenium catalysts significantly enhance ammonia production	[59]
K/Ru/TiO_2−*x*_H_*x*_	300 W Xenon lamp		112.6 μmol g^−1^ h^−1^		Enhanced hydrogen spillover and energy confinement at the interface	[60]
Fe nano-necklace/TiO_2−*x*_H_*x*_		5–10 W/cm^2^	1, 939 ppm (1 atm); 19,620 ppm (10 atm)		Dual temperature zone from the local heating of Fe	[61]
N_2_ fixation in presence of water (N_2_ + 6H^+^ + 6e^−^→2NH_3_)						
Au-Ru core antenna	Xenon lamp at the full spectrum	400 mW/cm^2^	101.4 μmol g^−1^ h^−1^ (2 atm N_2_)	0.21 % at 350 nm	Near electric field on the surface of Ru	[53]
Au/UiO-66	Xenon lamp with 400 nm long pass filter	100 mW/cm^2^	360 μmol g^−1^ h^−1^ (1 atm N_2_)	0.59 % at 520 nm	Large surface area for gas adsorption	[58]
Cu_96_Fe_4_	Xenon lamp	250 mW/cm^2^ (320–780 nm)	342 μmol g^−1^ h^−1^;	0.13 % at 535 nm	Valence state and coordination number of Fe increase	[62]
		200 mW/cm^2^ (400–780 nm)	242 μmol g^−1^ h^−1^
N_2_ fixation in presence of water and a sacrificial agent						
Au/TiO_2_ nanosheets	Xenon lamp at 420 nm cutoff filter		78.6 μmol g^−1^ h^−1^	0.82 % at 550 nm	Oxygen vacancy for N_2_ chemisorption	[54]
Au nanorod/CeO_2_	808 nm laser	8 W/cm^2^	114.3 μmol g^−1^ h^−1^		Oxygen vacancy for N_2_ chemisorption	[55]
	Broadband	100 mW/cm^2^	25.6 μmol g^−1^ h^−1^
Au embedded in hollow carbon nitride sphere	Xenon lamp at 420 nm cutoff filter		783.4 μmol g^−1^ h^−1^		Nitrogen vacancy for nitrogen activation	[57]
Au/g-C_3_N_4_	Xenon lamp at 420 nm cutoff filter		93 μmol g^−1^ h^−1^		Nitrogen vacancy for nitrogen activation	[56]

### 3.2 H_2_ production

H_2_ is not only a ubiquitous reducing agent for a multitude of reactions in heterogeneous catalysis and organic/pharmaceutical synthesis, but also a promising carbon-free fuel for vehicles and a high-density storage technology [64]. Many renewable applications try to split water into hydrogen and oxygen via photocatalysis, electrocatalysis, or photoelectrocatalysis [65–68]. The first instance of hydrogen evolution through direct light-driven water splitting dates back to 1971, when Fujishima and Honda discovered that UV irradiation on the surface of TiO_2_ enabled the direct photocatalysis of H_2_O into H_2_ and O_2_ [69]. Since then, scientists have explored various strategies to engineer semiconductor materials to achieve high yields of green hydrogen from solar energy. To achieve solar-to-hydrogen efficiency that is competitive with other hydrogen generation methods, a theoretical energy efficiency of 10 % would require a single absorber with a band gap smaller than approximately 530–600 nm [70]. The first milestone was achieved by Moskovits and co-workers, who used a Au-TiO_2_-Pt/Co thin-film device for photoelectrochemical water splitting to demonstrate the proof of concept that plasmon-induced hot carriers can be extracted to drive water splitting [71, 72]. Recent advancements have focused mainly on the fundamental aspects of extracting hot electrons or hot holes to facilitate both quantum efficiency and energy efficiency [73–75]. Many demonstrations of utilizing composite heterostructure semiconductors with plasmonic antennas to enhance visible light absorption are discussed in detail in ref. [15, 76]. Challenges such as constructing appropriate multiple Schottky junctions between metal and semiconductor for efficient charge separation and understanding the multiple electron coupling process in aqueous-phase reactions need to be further investigated.

Several reactions are used to produce hydrogen in industry, including ammonia decomposition, cracking of fossil fuels, methane reforming, and the Claus process. These methods generally require harsh operating conditions (high temperatures and pressures with transition metal catalysts) [77]. Recent progress in plasmonic photocatalysis suggests that non-thermal hot-carrier-driven reactions can be used for the production of hydrogen from a variety of sources with improved selectivity and lower activation barriers at mild conditions under visible light irradiation.

**Figure 3:**
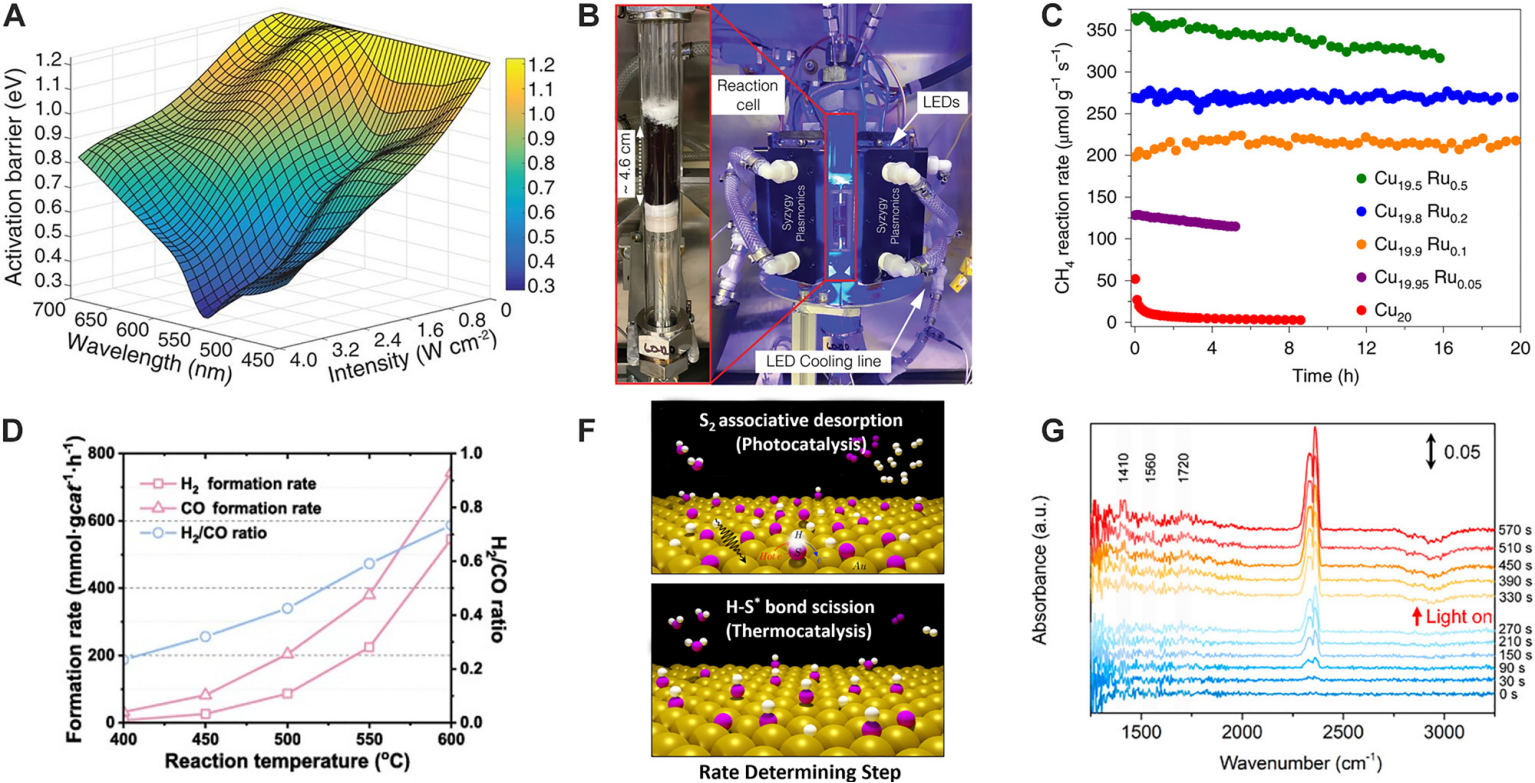
Plasmonic gas-phase catalysis for H_2_ generation. (A) Apparent activation energies under different wavelengths and power densities on a Cu-Ru surface alloy antenna reactor for NH_3_ decomposition (2NH_3_ → 2N_2_ + 3H_2_). Adapted from ref. [78] with permission. Copyright 2018 The American Association for the advancement of science. (B) 3D reactor to scale up the Cu-Ru and Cu-Fe catalysts in gram scale for ammonia decomposition with LED light. Adapted from ref. [79] with permission. Copyright 2022 The American Association for the advancement of science. (C) Stability test of CuRu for methane dry reforming (CH_4_ + CO_2_ → 2CO + 2H_2_). Adapted from ref. [80] with permission. Copyright 2020 Springer Nature. (D) Production rate and selectivity evolution with varying temperature of Au-Pt/P25 for photothermal catalysis. Adapted from ref. [81] with permission. Copyright 2022 Elsevier Inc. (E) Reaction mechanism of Au on SiO_2_ for direct photocatalytic and thermocatalytic H_2_S decomposition. Adapted from ref. [31] with permission. Copyright 2022 American Chemical Society. (F) *In-situ* FTIR measurement to detect the intermediate species during photochemical transformation on CuZn alloy for methanol steam reforming (CH_3_OH + H_2_O → 3H_2_ + CO_2_). Adapted from ref. [10] with permission. Copyright 2021 American Chemical Society.

Zhou, L., et al. reported Cu-Ru surface alloy antenna-reactors for ammonia decomposition (2NH_3_ → 2N_2_ + 3H_2_) [78]. Photocatalytic reactivity under white light illumination on the surface of Cu-Ru can be 1–2 orders of magnitudes higher than the reactivity from thermocatalysis at comparable temperatures. The energy efficiency for converting from light to chemical energy can be as high as 18 %. The apparent activation energies of the reaction under different wavelengths and power densities of light can decrease from 1.2 eV to 0.2 eV (Figure 3A), suggesting that hot carriers can help tailor the rate-determining step of this reaction, which shifts from the associative desorption of N_2_ in thermocatalyis to the N–H bond scission steps, therefore increasing the activity and reducing the activation barrier. Using the same synthetic techniques, the research team also compared Cu-Fe and Cu-Ru under similar light illumination conditions and found comparable performance. Originally Fe is famous for the strong adsorption with nitrogen species and can form FeN_*x*_, thereby deactivating the active centers. However, hot carriers can remove the surface N_*x*_ species and facilitate the desorption of NH_3_, turning Fe into an active center for photocatalytically decomposing the NH_3_. Additionally, the authors were able to change the light source from a laser to LED while scaling the reaction by 3 orders of magnitude (Figure 3B) and maintaining an energy efficiency of more than 10 % [79].

Methane can be utilized to extract synthesis gas (carbon monoxide and hydrogen) and has gained increased attention in light of abundant methane sourced from shale gas and tight oil [82]. Methane enables hydrogen production from direct cracking, steam reforming by reacting with water, dry reforming with CO_2_, and oxidation. Dry reforming is the most environmentally-friendly strategy as it reforms two greenhouse emission gases into CO and H_2_ but it generally requires high operating temperatures (700–1000 °C). Zhou., L., et al. incorporated a single atom Ru into a Cu nanoparticle by the co-precipitation method and were able to drive methane dry reforming (CH_4_ + CO_2_ → 2CO + 2H_2_) under visible light irradiation with decent stability over 50 h without coking [80]. Several ratios of Cu-Ru were tested. However, most of them were deactivated due to coking on the surface, as shown in Figure 3C. Only Cu_19.8_Ru_0.2_ and Cu_19.9_Ru_0.1_ exhibited both reactivity and selectivity for over 20 h. The authors further confirmed that Ru existed in the form of a single-atom feature by utilizing infrared CO adsorption measurements combined with a DFT + D3 calculation. By comparing the reactivity and selectivity of thermocatalysis, they suggested that the plasmon-induced desorption assists in tailoring the pathway, and the single-atom Ru center feature prevents the adjacent carbon landing sites, thereby reducing coking. Furthermore, Zhang., Z., et al. reported a reaction rate of 85.38 mmol g_cat_^−1^ h^−1^ and 201.92 mmol g_cat_^−1^ h^−1^ for H_2_ and CO, respectively, using the plasmonic photothermal effect on their Pt-Au/P25 composite catalysts [81]. The results presented in this study demonstrate a 3-fold increase in reactivity compared to the reaction in the absence of light. Furthermore, the reactivity and selectivity were observed to be stable for over 15 h. The selectivity was higher at higher temperatures for both photo- and thermo-catalysis, indicating the dominance of the photothermal effect in the reaction (Figure 3D). Additionally, the authors used *in-situ* FTIR measurements to identify intermediate species of CH_*x*_, HCOO^*^, and H^*^ on the surface of their catalysts. The HCOO^*^ species was found to be the most important in driving the reaction towards the production of CO and H_2_ by decomposition. Based on these findings, a detailed mechanism was proposed in which plasmon-induced hot carriers on the surface of TiO_2_ help convert CH_4_ into CH_*x*_, while the Pt surface cleaves CO_2_ and spillovers the oxygen to the interface to facilitate the formation of HCOO^*^. Takami., D., et al. reported the activation of the reaction at the low temperature of 200 °C over a plasmonic Ni/Al_2_O_3_ catalyst, maintaining a high conversion of CH_4_ and CO_2_ (20 %) [83]. They recognize that the metallic Ni that support LSPR is the key to maintain the high conversion at low temperatures.

Other avenues for sustainable hydrogen production from plasmonic photocatalysis include hydrogen sulfide decomposition (H_2_S → H_2_ + S) and methanol steam reforming (CH_3_OH + H_2_O → 3H_2_ + CO_2_). Lou., M., et al. used plasmonic Au nanoparticles supported on silica without any external heating source to catalyze one-step direct H_2_S decomposition, a potential alternative for the industrial two-steps Claus process to obtain sulfur and H_2_ (Figure 3E) [31]. The results of ground and excited-state first-principle calculations, combined with partial-pressure dependent reactivity and micro-kinetic analysis, revealed that plasmon-induced hot electrons on the surface of Au nanoparticles shift the rate-determining steps from H–S^*^ bond scission in dark conditions to S_2_ associative adsorption under light irradiation. Luo., S., et al. reported a H_2_ production rate of 328 mmol g_cat_^−1^ h^−1^ for methanol steam reforming on a plasmonic CuZn alloy, with a light energy conversion efficiency of 1.2 % at 7.9 suns irradiation from a solar simulator [10]. The *in-situ* diffuse reflective infrared Fourier transform spectra (DRIFTS) confirmed the key intermediate species on the copper-based catalysts are CH_*x*_O and HCOOH. The light illumination helps increase the concentration of the intermediates, thereby improving reactivity (Figure 3F). Gas-phase H_2_ production publications are summarized in Table 2. Incorporation of these state-of-art experiments into actual industrial production requires parameter optimization and large-scale photoreactor design. Indeed, several industries and start-ups are now addressing 3D design of reactors to better utilize all the photons for photochemical transformations and to improve flow of the gas to efficiently penetrate the reactor. Although the high-efficiency conversion of H_2_ generation developed so far in the field seems to facilitate the possibility of industrial-level production, fundamental studies on understanding the dynamics of hot carriers and how to extract them into chemistry are essential to increase quantum and energy efficiency. Moreover, renewable light sources such as solar energy have a rather complex wavelength distribution, making the study on extracting them more challenging than the exciting results achieved using monochromatic light sources such as lasers and LEDs.

**Table 2:** Summary of gas-phase photocatalytic H_2_ production.

Nanostructure	Light source	Power density	H_2_ rate	Quantum/energy efficiency	Highlights of the work	Reference number
Ammonia decompostion: N_2_ + 3H_2_ → 2NH_3_						
Cu-Ru surface alloy	Supercontinuum laser with a bandpass filter	9.6 W/cm^2^	1200 μmol g^−1^ s^−1^	18 % (energy); 33.5 % (quantum)	Hot-electron tailored to the N–H bond scission. Activation energies range from 1.2 to 0.2 eV under illumination.	[78]
Cu-Fe	Supercontinuum visible laser	200 mW	466 μmol g^−1^ s^−1^		Photoreactor with LED light to scale up the reaction.	[79]
	470 nm LED	180 W	14 g/day	15.6 % (energy)
Methane dry reforming: CH_4_ + CO_2_ → 2CO + 2H_2_						
Cu-Ru	Supercontinuum laser	19.2 W/cm^2^	550 μmol g^−1^ s^−1^	15 % (energy)	Single-atom Ru sites help alleviate the coking and long-term stability	[80]
Au-Pt/P25	Xenon lamp	4.6 W/cm^2^	201.92 mmol g^−1^ h^−1^		*In-situ* FTIR for the mechanism study	[81]
Ni/Al_2_O_3_	Xenon lamp		1.2 mmol h^−1^		20 % conversion rate under 473 K low temperature	[83]
H_2_S decomposition: H_2_S → H_2_ + S						
Au/SiO_2_	Supercontinuum visible laser	13 W/cm^2^	5 μmol g^−1^ s^−1^	1.8 % (quantum)	One-step direct decomposition. Light-induced rate-determining step shift	[31]
Methanol steam reforming: CH_3_OH + H_2_O → 3H_2_ + CO_2_						
CuZn alloy	Solar simulator AM 1.5 G	788 mW/cm^2^	328 mmol g^−1^ h^−1^	1.2 % (energy); 6.5 % (quantum)	Zn serves atoms serve as active sites and charge transfer center	[11]

### 3.3 CO_2_ reduction

Reaction pathways with multiple electron transfer processes can potentially be used to reduce CO_2_ in the presence of water or H_2_ to turn CO_2_ into value-added chemical products [84]. The metal-molecule interface of the chemical transformation can be modified via optical near-fields, hot carrier generation and multiplication [85], localized photothermal effects and gradients [86, 87], and the construction of active centers. Control of these parameters allows for coupling optical energy into different products with high selectivity, allowing for tailor-made catalysts that target particular chemical reaction pathways.

Yu., S., et al. investigated the mechanism of C–C coupling on the surface of Au with CO_2_ in the presence of H_2_O. The selectivity was highly tailored by both the wavelength and power density of the incident continuous wave (CW) lasers: High-intensity resonant illumination of interband transitions at 488 nm showed preferred selectivity to ethane (corresponding to a multi-electron harvesting process) while excitation at 532 nm pushed the reaction towards methane production [7]. The team found that the observed selectivity trend can be attributed to the nature of higher light intensity and photon energy, which can result in a multi-carrier process. They also observed that the reactivity and selectivity follow the Poisson statistics of electron harvesting. The team also investigated the same reaction on Ag plasmonic nanoparticles at a single-particle level by employing *in-situ* Raman spectroscopy. They detected C_1_–C_4_ product evolutions with multiple intermediate species with visible light irradiation at mild conditions [6, 88]. Trace amounts of rich catalog of multiple carbon species were generated through a multi-photon excitation process during plasmon decay. However, the detailed mechanism of this process and how to regulate the selectivity towards long carbon-chain products require further investigation and development. Abundant reports have been published of hybrid plasmonic nanoparticles with semiconductor materials or MOFs to enhance the light–matter interaction and regulate the selectivity of the major products towards CH_4_ [89, 90], CO [91–93], CH_3_OH [94], HCOOH [63, 95, 96], and C_2_H_6_ [97], among others, by taking advantages of the unique electronic structure and vacancy sites on the metal oxide semiconductor or the large surface area of MOF. Understanding the mechanism and manipulating multi-electron processes through nanostructure engineering remains a future challenge of the field given the complex possible reaction pathways.

**Figure 4:**
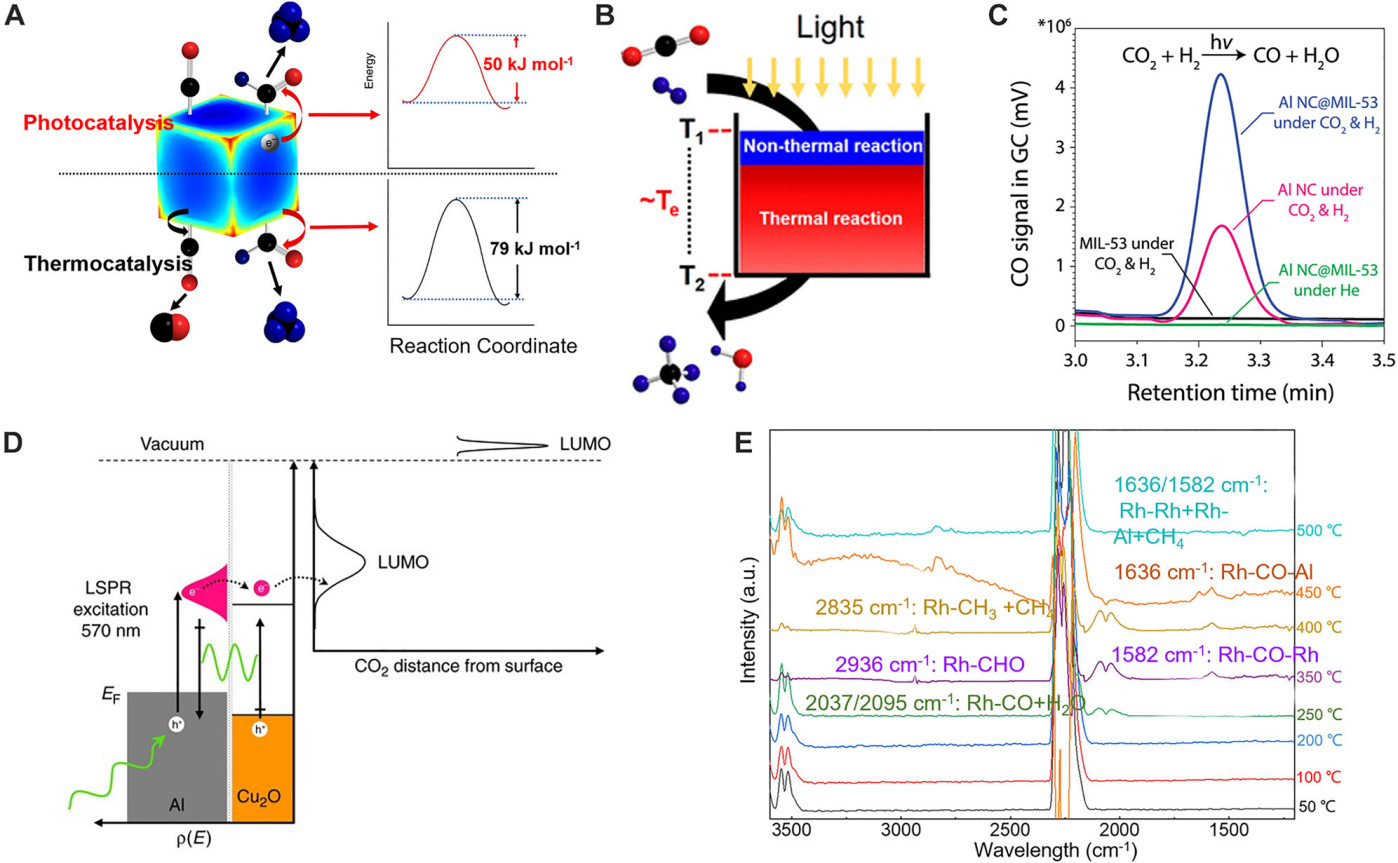
Plasmonic gas-phase CO_2_ hydrogenation. (A) Activation energies and selectivity of CO_2_ hydrogenation towards methane on Rh/Al_2_O_3_ catalysts are tailored by light compared to the reaction in the dark. Adapted from ref. [9] with permission. Copyright 2017 Springer Nature. (B) CO_2_ methanation reaction on Rh/TiO_2_ catalysts utilizing both thermal and non-thermal effects with the temperature gradient in the catalyst bed. Adapted from ref. [98] with permission. Copyright 2018 American Chemical Society. (C) Al@MIL-53 for enlarged surface area for gas uptake and enhanced reverse water-gas shift reaction. Adapted from ref. [99] with permission. Copyright 2019 The American Association for the advancement of science. (D) Schematic of hot-electron induced chemistry at Al-Cu_2_O interface for reverse water-gas shift reaction. Adapted from ref. [5] with permission. Copyright 2017 Springer Nature. (E) *In-operando* FTIR measurement of reaction steps under different temperatures for photothermal CO_2_ methanation on a Rh/al catalyst. Adapted from ref. [8] with permission. Copyright 2021 American Chemical Society.

Gas-phase CO_2_ hydrogenation is another approach to use renewable light energy to produce valuable chemical products. Rather than the multiple electron transfer process as in the presence of water, gas-phase CO_2_ hydrogenation mainly has two reaction pathways under room pressure: CO_2_ methanation (CO_2_ + 4H_2_ → CH_4_ + 2H_2_O) and reverse water-gas shift reaction (CO_2_ + H_2_ → CO + H_2_O). Zhang., X., et al. observed the non-thermal effect on Rh/Al_2_O_3_ catalysts under visible LED illumination driving reaction towards CH_4_ with selectivities >86 % and >98 % under illuminations of blue (400 nm) and ultraviolet (365 nm) light, respectively [9]. Compared to thermocatalysis, plasmonic photocatalysis can selectively activate the C-O bond in CHO* intermediates by hot electrons with an apparent activation energy decrease (Figure 4A). The authors also reported a mechanistic study of a Rh/TiO_2_ catalyst, showing the selective methanation of CO_2_ through synergistic photothermal and hot-carrier effects (Figure 4B) [98]. The photothermal effect and the plasmon-mediated non-thermal effect (Figure 4B, iii) can be distinguished and quantified by measuring the top and bottom temperatures of the catalyst bed and combining them with a model. In addition, their DFT calculation found that CHO^*^ is a critical intermediate for CO_2_ methanation in the presence of H_2_. However, increasing the hydrogen partial pressure would inhibit the injection of hot electrons into the orbital of CHO^*^. Robatjazi., H., et al. designed a sustainable aluminum-based core–shell structure of Al@MIL-53 and Al@Cu_2_O to drive the reverse water-gas shift reaction by increasing the physisorption of the gas, and extracting hot electrons from Al, respectively (Figure 4C and D) [5, 99]. Al@MIL-53 enables up to eight times larger CO_2_ uptake than Al nanocrystals, resulting in a four times higher reactivity towards the reverse water-gas shift reaction (shown in Figure 4C). In Al@Cu_2_O, the small Schottky barrier between Al and Cu_2_O (shown in Figure 4D) facilitates the separation of hot carriers generated from the Al core. This results in a regulation of selectivity towards the reverse water-gas shift reaction under visible-light illumination. Additionally, this boosts the EQE to two orders of magnitude higher than bare Cu_2_O and one order of magnitude higher than Al nanoparticles. Fu., G., et al. designed a Rh/Al photothermal catalyst from colloidal synthesis with Al powders and RhCl_3_ precursor, capable of regulating CO_2_ hydrogenation towards methanation with nearly 100 % selectivity under a concentrated simulated solar irradiation at 11.3 W/cm^2^, resulting in a 550 mmol g^−1^ h^−1^ reaction rate [8]. The *in-operando* Temperature-Programmed FTIR analysis identified the surface CO species on Rh is the key intermediate steps to drive the reaction towards the methanation process (Figure 4E). Table 3 outlines the relevant citations for gas-phase CO_2_ hydrogenation reactions. In our opinion, further research should focus on resolving the multiphoton and electron processes under high light intensity and photon energy in both aqueous and gas-phase CO_2_ reduction. Additionally, constructing active centers with different electronic structures for the various carbon-related species to accumulate and desorb can help regulate the selectivity of CO_2_ conversion into valuable molecules. Even for extensively studied gas-phase reactions such as reverse water-gas shift and methanation, understanding the time resolution of the intermediate steps is important to determine how to optimize the lifetime of plasmon excitation and decay to improve conversion.

**Table 3:** Summary of gas-phase photocatalytic CO_2_ hydrogenation.

Nanostructure	Light Source	Power density	Selectivity	The highlight	Reference
				of the work	number
Rh/Al_2_O_3_	UV (365 nm) LED	3 W/cm^2^	>98 % to methane	Activation energy decrease and selectivity control to methanation	[9]
	Blue (400 nm) LED		>86 % to methane
Rh/TiO_2_	UV (365 nm) LED	2.24 W/cm^2^	>98 % to methane in both light and dark	46 % quantum efficiency at 350 °C surface temperature	[98]
	Blue (400 nm) LED	3.55 W/cm^2^
Al@Cu_2_O	Supercontinuum laser	10 W/cm^2^	>99.3 % toward CO	3 % quantum efficiency. Hot electrons in the Cu_2_O shell drive the transformation	[5]
Al@MOF	Supercontinuum laser	350 mW	CO	Enhanced gas uptake increases the reactivity	[99]
Rh/Al	Solar simulator	11.3 W/cm^2^	Nearly 100 % selectivity towards methane.	CH_4_ production rate at 550 mmol g^−1^ h^−1^ under a concentrated solar simulated source (11.3 W/cm^2^) irradiation. And *In-operando* FTIR measurement for multiple steps activation and conversion.	[8]

## 4 Electromagnetic and quantum predictions of photochemical transformations

### 4.1 Wavelength-dependent photochemical transformations

For intermediate-sized nanoparticles (>5 nm and <100 nm), Mie theory and Gans theory are often employed to model the dielectric constant of a metal to analytically determine the absorption, scattering, and extinction spectra of metal nanoparticles with varying shapes, sizes, compositions, and dielectric environments. These methods are described in detail in several excellent review papers [28, 100]. Numerical methods such as finite-difference time-domain (FDTD), boundary element method (BEM), and finite element methods (FEM), can also be used to simulate and calculate the optical properties of the plasmonic nanostructure with high accuracy by solving Maxwell’s equations in three dimensions, depending on the dimension, geometry, symmetry, etc. In addition to simulating spectra and correlating these with experimental extinction, diffuse reflectance, dark-field scattering, etc., classical or semi-classical electromagnetic calculations offer us a tool to distinguish the mechanism of various reaction pathways and to interpret wavelength-dependent reactivity of plasmonic photocatalysts. An early example of utilizing this tool to distinguish the hot electrons in metal includes Zheng, B. et al., in which they fabricated a Au-TiO_2_ device and measured the photocurrent with the Schottky contact and Ohmic contact between Au and TiO_2_ interface [101]. The authors exploit the fact that only the plasmon-induced hot electrons have sufficient energy to overcome the Schottky barrier. The study confirmed that hot carrier generation within a plasmonic metal is directly correlated to the volume integration of |*E*|^2^ within the electron mean free path of the metal. The same idea can also be applied to Al@Cu_2_O core–shell nanoparticles for reverse water-gas shift, Al-Pd antenna-reactors for H_2_ dissociation and C–F activation, and solvated electrons from Au and Ag nanoparticles [5, 102–104]. There are many publications which correlate absorption or extinction cross-sections with wavelength-dependent reactivity, especially when the classic absorption cross-section is very close to its extinction cross-section in Au nanoparticles [31, 53, 58, 62]. However, the portion of the energy or charge transfer process directly taking part in the chemistry is not clear in this paradigm. Yuan., L., et al. developed a model based on the semi-classical hot electron theory applied to aluminum plasmonic nanocrystals from a previous report [34, 105]. The interband hot electrons were accounted for by including the contribution of the interband transition to the imaginary part of the permittivity in the |*E*|^2^ integration. Besides the peak originating from the plasmon modes, only the hot-electron cross-section follows the increasing trend at 700–800 nm, which matches well with the experiment. The strong quantum confinement due to the small size or features of nanoparticles also give rise to phonon-assisted Landau damping (intraband transition) hot carriers. These are usually considered a bonus to the classic absorption cross-section, but one can consider adding the corresponding term into the dielectric constant of the metal just like the Drude electron term and Lorentzian oscillators for interband transition (or another model such as Brendal–Bormann, and quantum mechanical model, etc.). [106, 107]. The semi-classical theory can easily decouple the hot electron cross-section from direct photoexcitation (interband transition), Landau damping (intraband transition), and photothermal effects by comparing the spectra with the wavelength-dependent reactivity or quantum efficiency.

Numerous challenges remain in electromagnetic calculations, such as taking into account the detailed picture of the charge transfer of hot electrons to the anti-bonding orbitals of the molecule (or hot holes to bonding orbitals). An illustrative example is the utilization of a Lorentzian function in hot carrier integration. This function effectively characterizes the energy position distribution of a molecular adsorbate, enabling hot carriers produced at specific energy levels to be injected into the adsorbates, consequently prompting the desired chemical reactions [79, 108]. Whether the reaction rate or external quantum efficiency should be chosen to correlate the calculated hot electron cross-section (or rate) depends on how the hot carriers impacts the conversion of molecules and whether it is one hot electron corresponding to one molecule or if carrier multiplication also contributes to the reaction [85, 109]. Moving forward, further developments in high-accuracy quantum chemistry calculations, ultrafast pump-probe measurements, and *in-operando* measurements of reaction intermediates will be needed to obtain accurate information on these processes.

## 5 Quantum mechanistic insights on plasmonic photocatalysis

To better understand the mechanisms underlying plasmon catalysis, considerable effort has been devoted to computationally predicting excited-state processes. Such calculations are necessary to explain plasmon creation and dephasing, as well as the excited-state photochemistry arising from the presence of hot carriers and non-equilibrium phonon distributions.

To describe light–matter interaction in nanoparticles, first-principles atomistic calculations based on density functional theory (DFT) and time-dependent DFT (TDDFT) can only simulate systems of relatively small sizes (typically nanoparticles of hundreds or few thousands of atoms). Still, they offer unique quantum-mechanical insights into the physical origin of plasmon dephasing and charge transfer [110–118]. The other extreme limit is that of a nanoparticle large enough that its plasmon dephasing and hot electron generation can be modeled from a simpler geometry by considering a semi-infinite metallic slab. Accurate computations were imperative in elucidating the contribution of *d* bands in noble metals to the production of hot electrons from surface plasmon polaritons [119, 120], as well as the impact of phonons and finite-size effects on plasmon damping [22].

A major challenge in predicting excited-state chemistry is to accurately address the interplay between local chemical environment and excited-state electronic and optical properties. Quantum mechanical, atomistic effects, and electron correlation beyond what is available with mean-field formalisms such as DFT must be included. There are a few directions that the community is exploring towards this goal. First, there has been qualitative success in the utilization of embedding techniques that treat chemically active sites with a high-level quantum chemistry theory, while the remainder of the nanoparticle (or approximate planar slab geometry) is treated approximately within DFT. For instance, embedding theory was recently used to elucidate the hot-electron-based splitting of H_2_ on an Au surface [121].

There are also numerous examples employing high-accuracy excited-state calculations to identify the potential transient negative ion (TNI) state to explain the reduction of energy barriers for key intermediate steps compared to a ground-state reaction pathway, helping to elucidate the unique properties of plasmon-induced chemistry [31, 108, 121].

In addition, theoretical approaches based on quantum electron dynamics (QED), wherein one explicitly includes the quantized photon modes together with interacting electrons treated within DFT or correlated wavefunction approaches, allows one to include strong electromagnetic fields when describing such chemical reactions [122]. Finally, emerging large-scale quantum many-body formalisms [123–126], such as those based on the GW approximation to the electronic self-energy [127] and the Bethe-Salpeter equation (BSE) [128], are poised to address (i) the coupling of light with plasmons and other possible excited states in the nanoparticles; (ii) the atomistic details of the reaction, including realistic surface reconstruction, dopant distributions, and adsorbate reaction coordinates; and (iii) the net results of these excitations into the overall chemical reaction, including any necessary quantum many-body interactions.

## 6 Summary and outlook

In this article, we introduced the unique features of plasmonic photocatalysis and potential opportunities to use renewable light energy to drive sustainable chemistry. We explored recent progress in three promising applications: plasmon-induced ammonia synthesis, gas-phase hydrogen generation, and CO_2_ reduction. The importance of these three applications cannot be understated, as they are critical for the sustainable production of food, fuel, and feedstock for organic/pharmaceutical synthesis. Electromagnetic theory and quantum chemistry are two toolboxes which provide unique insight into light–matter interactions and surface-adsorbate interfaces. Experiment and theory are both necessary to determine the underlying mechanisms in plasmonic photocatalysis and for further optimization of the light–matter and molecule-active center interactions to synergistically increase the yield and maximize the selectivity for future applications and industrialization.

Future work in the field will include more advanced *in-situ* and *in-operando* measurements such as employing optically-coupled environmental transmission electron microscopy to understand light-induced phase transformations beyond hydrides [129–131] on the sub-nm scale. FTIR and Raman spectroscopy can also be employed to identify key intermediates on the surface of catalysts. High-accuracy quantum mechanical simulation methods for both light–matter interactions and excited-state reaction pathways are also expected to be developed. Resolving the time-scale of critical intermediate species and plasmon lifetime on different surfaces remains a significant challenge. The field should focus on obtaining a detailed picture of the photochemistry, especially for small molecules with fewer side reactions, through quantitative measurements. Subsequently, researchers need to probe and control the more complicated chemistry, such as the Fischer–Tropsch synthesis, by understanding the multiphoton and hot-carrier processes. Looking forward, the use of earth-abundant materials for catalysts, renewable light sources such as solar light and LED illumination, recycling of noble metal catalysts, and scaling reactor sizes are all critical developments toward the goal of sustainable chemistry. The use of green energy sources in the chemical industry will be vital. Taking the steel industry as an example, both using green hydrogen for CO_2_ reduction and developing composite materials with the necessary mechanical and corrosion properties would significantly reduce the industries carbon footprint and support the development of sustainable chemistry.
